# Variations in Leaf Functional Traits of *Pseudotsuga sinensis* Across Forests With Varying Levels of Rocky Desertification

**DOI:** 10.1002/ece3.70916

**Published:** 2025-02-01

**Authors:** Wangjun Li, Wanchang Zhang, Tu Feng, Dongpeng Lv, Shun Zou, Bin He, Xiaolong Bai

**Affiliations:** ^1^ Guizhou Key Laboratory of Plateau Wetland Conservation and Restoration Guizhou University of Engineering Science Bijie Guizhou China; ^2^ Key Laboratory of Digital Earth Science, Aerospace Information Research Institute Chinese Academy of Sciences Beijing China; ^3^ International Research Center of Big Data for Sustainable Development Goals Beijing China; ^4^ Key Laboratory of Ecological Microbial Remediation Technology of Yunnan Higher Education Institutes Dali University Dali Yunnan China

**Keywords:** functional traits, leaf anatomy, leaf morphology, *Pseudotsuga sinensis*, rocky desertification

## Abstract

*Pseudotsuga sinensis* is a distinctive plant species endemic to China, predominantly found in areas affected by varying degrees of rocky desertification. Despite its wide distribution, the physiological mechanisms underlying its adaptation to harsh environments remain unclear. In this study, we investigated 16 leaf traits, including the morphological, anatomical, and chemical characteristics of the leaves of *P. sinensis* across forests with mild, moderate, severe, and extremely severe rocky desertification to elucidate the adaptive strategies of *P. sinensis* in response to arid conditions and nutrient‐poor soils. Our findings revealed that *P. sinensis* leaves from forests with mild and moderate rocky desertification exhibited higher specific leaf area (SLA) and magnesium concentrations but lower leaf dry matter content (LDMC), abaxial epidermis thickness, and adaxial epidermis thickness than in those from forests with severe and extremely severe desertification. Principal component analysis indicated that forests with mild to moderate desertification employ resource acquisition strategies characterized by greater SLA and magnesium concentrations than those in forests with severe and extremely severe desertification. In contrast, forests with severe to extremely severe desertification adopted resource‐conserving strategies, as evidenced by higher LDMC, epidermal thickness, and calcium concentrations than those in forests with mild to moderate desertification. The N:P ratio of *P. sinensis* across all desertification levels was consistently below 14, suggesting nitrogen limitation in *P. sinensis* in regions with rocky desertification. Thus, these results provide valuable reference for guiding vegetation restoration under degraded habitats.

## Graphical abstract

This study investigates 16 leaf traits—encompassing morphological, anatomical, and chemical characteristics—of *Pseudotsuga sinensis* across forests with mild, moderate, severe, and extremely severe rocky desertification. The objective was to elucidate the adaptive strategies of *P. sinensis* in response to arid conditions and nutrient‐poor soils.

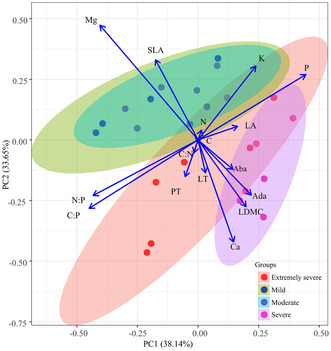

## 1 Introduction

*Pseudotsuga sinensis*, a member of the genus *Pseudotsuga* in the family Pinaceae, is primarily distributed across the Yunnan, Guizhou, Sichuan, Hubei, and Hunan provinces of China (Xiong et al. 2017; Wen 2022). This species is a relic from the Tertiary period and is considered endangered and endemic to China (Xiong et al. 2017). The distribution of *P. sinensis* is sporadic throughout China (Nong et al. 2024). The pure forests of this species are found in limited locations, including Badagongshan in Hunan Province and Baimianshui provincial nature reserves in Meitan County (210 ha; Hu 2015) and Weining County in Bijie City (373.2 ha; He et al. 2021) in Guizhou Province. Over the last few decades, excessive deforestation has led to an increasingly severe destruction of *P. sinensis* forests (Sun et al. 2003). In addition, climate change threatens the distribution of *P. sinensis* by affecting its habitat migration (Nong et al. 2024). Natural factors and human interference, combined with low seed setting, seed germination, and seedling survival rates, have led to a slow regeneration of *P. sinensis* communities under natural conditions, thus causing a serious challenge in the maintenance of *P. sinensis* populations (Sun et al. 2003; Zhang, Guo, and Jiang 2004; Meng et al. 2008). Therefore, a study of the physiological and ecological mechanisms governing the adaptation of *P. sinensis* populations to environmental change is urgently needed.

Hitherto, research on *P. sinensis* has primarily focused on its community structure (Li et al. 2021), population dynamics (Wen 2022), phytogeographical distribution (Li et al. 2019; He et al. 2022; Nong et al. 2024), germplasm conservation and utilization strategies (Sun et al. 2003), chemical constituents (Yi, Zhang, and Li 2002), and genomic characteristics (Li, Feng, et al. 2023). In contrast, few studies have focused on the effects of environmental factors on the distribution of *P. sinensis* . Li et al. (2024) reported that altitude is the dominant environmental factor affecting the distribution of *P. sinensis* in the Sichuan Province. Nong et al. (2024) reported that rainfall, altitude, and temperatures during the coldest month are the main environmental factors affecting the distribution of *P. sinensis* in China. In addition, researchers have reported that *P. sinensis* exhibits adequate growth in both fertile acidic soil and impoverished soil in habitats with rocky desertification (Li and Xie 2015; Li, He, et al. 2023). However, the physiological mechanisms underlying its adaptation to environmental changes remain largely unknown.

The rocky desertification area in Guizhou Province, Southwest China, is the largest and most representative of its kind (Su, Zhu, and Xiong 2002; Liu et al. 2011). In these forests with karst rocky desertification, the landscape is characterized by a relatively high degree of rock exposure, thin soil layers, low vegetation cover, and poor soil nutrients and water retention capacity. These conditions lead to prolonged drought and nutrient stress in plants (Su, Zhu, and Xiong 2002; Li, Tan, and Wang 2005; Jiang, Lian, and Qin 2014; Xiong and Chi 2015). Despite these harsh conditions, *P. sinensis* is concentrated in mountainous forests that have experienced severe rocky desertification (Hu 2015; He et al. 2021). Therefore, elucidating the mechanisms underlying the adaptation of *P. sinensis* to these fragile habitats with rocky desertification is important.

Plant functional traits, including morphological, physiological, and phenological characteristics, are closely related to plant adaptability (Violle et al. 2007). Leaf morphological traits, such as leaf area (LA), are indicative of light resource acquisition and gas exchange capacity, with plants often producing smaller leaves in water‐scarce environments than in water‐rich environments (Ackerly et al. 2002; Cornelissen et al. 2003). Specific LA (SLA), which represents the light capture area per unit mass of dry matter, is generally lower in resource‐poor environments than in resource‐rich environments (Reich et al. 1998). Leaf dry matter content (LDMC), which is related to nutrient retention capacity, is typically higher in resource‐limited habitats than in resource‐rich habitats (Cornelissen et al. 2003; Yang et al. 2014).

Regarding leaf anatomical traits, plants often develop relatively thick leaves and tissues to enhance their water storage and utilization efficiencies (Liu, Liu, and Guo 2011; Zhang et al. 2019, 2020). N, P, and K are critical for plant metabolism, including protein and ATP syntheses, and osmotic regulation (Evans 1989; Lambers, Chapin, and Pons 1998; Roelfsema and Hedrich 2005). Studies have indicated that as the degree of rocky desertification increases, the concentrations of these nutrients in plant leaves decrease (Bai et al. 2024; Liu and Wang 2024). Conversely, Ca, which is essential for cell wall stability and the formation of cellular structures (White and Broadley 2003), and Mg, which is required for photosynthesis and plant development (Chen et al. 2018), are often present at relatively high levels in plants from forests with karst rocky desertification (Bai et al. 2024; Liu and Wang 2024).

The C:N:P ratios in plant leaves provide insights into survival strategies and nutrient limitation (Koerselman and Meuleman 1996; Elser, Sterner, et al. 2000; Güsewell 2004). Specifically, the C:N and C:P ratios are the critical indicators of the rates of plant growth and the efficiency of N and P utilization (Herbert, Williams, and Rastetter 2003; McGroddy, Daufresne, and Hedin 2004; Wu et al. 2020). The N:P ratio is used to assess nutrient limitation, with thresholds indicating N limitation (N:P < 14), P limitation (N:P > 16), or the limitations of both nutrients (14 <N:P < 16) (Koerselman and Meuleman 1996). Previous studies have shown that the growth of plants in the family Pinaceae is often limited by N (Hu et al. 2007; Liu and Dong 2020). However, it remains unclear whether the growth of *P. sinensis* is limited by N, P, or both.

In the present study, we tested the leaf morphological, anatomical, and physiological traits of *P. sinensis* across forests with varying degrees of rocky desertification (mild, moderate, severe, and extremely severe) to elucidate the different adaptive strategies employed by *P. sinensis* in response to varying levels of desertification. In response to the intensification of rocky desertification, increased light exposure, and decreased soil water content, plants can increase the retention time of water and nutrients in the body by reducing their SLA but increasing their LDMC and epidermal thickness to adapt to harsh environments (Bai et al. 2024). Therefore, we hypothesized that the SLA of *P. sinensis* leaves would increase with an increase in the degree of rocky desertification and that their dry matter content, abaxial epidermis thickness (Aba), and adaxial epidermis thickness (Ada) would decrease with an increase in the degree of rocky desertification. In addition, with the intensification of the degree of rocky desertification, the concentrations of available soil nutrients decrease and those of Ca increase (Ji, Li, and Deng 2009). Therefore, we further hypothesized that K and Mg concentrations in *P. sinensis* leaves would decrease, but their Ca concentrations would increase with the intensification of the degree of rocky desertification. Previous studies have shown that the growth of plants in habitats with rocky desertification is generally limited by P (Liu, Zhong, and Ni 2019; Yang et al. 2020). Therefore, we also hypothesized that the growth of *P. sinensis* in forests with different degrees of rocky desertification is limited by P.

## 2 Materials and Methods

### 2.1 Study Area

This study was conducted in the *P. sinensis* Nature Reserve, located in Bijie City, Guizhou Province, southwest China (103.93° E–104.26° E, 26.54° N–26.76° N). The elevation of the study area ranges from 1800 to 2450 m above sea level. The region is characterized by a subtropical monsoon climate, with a mean annual temperature of 10.5°C and annual precipitation totaling 1000 mm. The predominant soil types in the area are yellow‐brown soil and latosol, with a pH value of 5.50 (He et al. 2021).

### 2.2 Sampling of *P. sinensis* Leaves

The sampling sites in this study were classified into four categories based on their degree of rocky desertification, as detailed in Table 1: mild (rock exposure rate: 30%–50%; vegetation coverage: 50%–70%; average soil thickness: 30–50 cm), moderate (rock exposure rate: 50%–70%; vegetation coverage: 30%–50%; average soil thickness: 20–40 cm), severe (rock exposure rate: 70%–90%; vegetation coverage: 10%–30%; average soil thickness < 20 cm), and extremely severe (rock exposure rate > 90%; vegetation coverage < 10%; average soil thickness < 10 cm) rocky desertification (Li, Dong, and Wang 2007).

**TABLE 1:** Characteristics of the sampling sites.

Characteristics	Mild rocky desertification	Moderate rocky desertification	Severe rocky desertification	Extremely severe rocky desertification
Elevation	2121 m	2008 m	1996 m	1872 m
Longitude	104°04′21″ E	104°04′23″ E	104°02′49″ E	104°04′52″ E
Latitude	26°37′23″ N	26°37′43″ N	26°36′29″ N	26°37′21″ N
Slope	27°	25°	35°	15°
Aspect	NW310°	E96°	SE134°	NE53°
Soil types	Yellow‐brown soil	Yellow‐brown soil	Yellow‐brown soil	Yellow‐brown soil
Rock types	Limestone	Limestone	Limestone	Limestone

**FIGURE 1:**
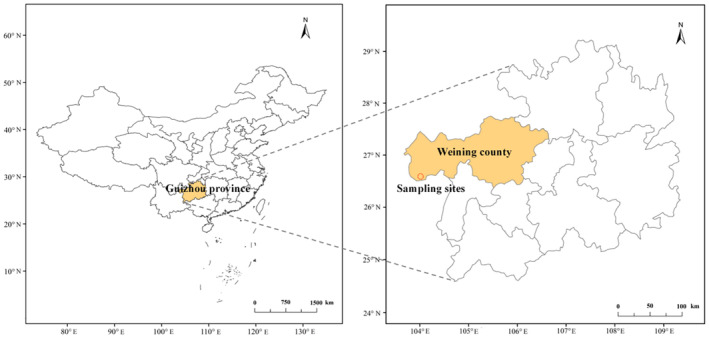
Location of the sampling sites.

We established 20 × 20 m quadrats for each forest type corresponding to these categories. Within each quadrat, we further subdivided the area into four 10 × 10 m subquadrats. *Pseudotsuga sinensis* specimens were selected near the center of both the 20 × 20 m and 10 × 10 m quadrats for leaf collection. For each tree, we used high‐pruning techniques to cut four branches in different directions and collected healthy, mature, and intact leaves. The leaf samples were placed in a sampling box and transported to the laboratory for subsequent trait measurements. Detailed information of the sampling sites is shown in Figure 1 and Table 1.

### 2.3 Trait Measurements

Leaf functional traits were measured following the methodologies outlined by Cornelissen et al. (2003). For each tree, 20 leaves were collected to assess LA. As *P. sinensis* leaves are flat, we measured their leaf width and length (cm) and leaf thickness (LT, μm) at the midpoint of each leaf using a Vernier caliper. LA was calculated by multiplying leaf width by leaf length, using the formula LA = leaf width × leaf length.

The leaf fresh weights of the 20 sample leaves from each tree were recorded using an electronic balance (precision: 0.0001 g), and the samples were then oven‐dried at 70°C for 48 h. Subsequently, leaf dry weight was measured using the same electronic balance. SLA (cm^2^/g) was calculated by dividing LA by leaf dry weight. LDMC (g/g) was calculated as the ratio of leaf dry weight to leaf fresh weight.

For the anatomical analysis, five leaf samples were sliced at the midpoint using a single‐sided blade. Each slice was examined under a binocular microscope (Leica DM2500; Leica, Wetzlar, Germany), and 5–7 cross‐sectional images were captured. Ada (μm), Aba (μm), and palisade mesophyll thickness (PT, μm) were measured using ImageJ Software (https://imagej.en.softonic.com/).

Dry leaf samples were ground to a fine powder using a crusher and sieved through a 60‐mesh screen. Leaf C (mg/g) and N (mg/g) concentrations were determined using a Dumas‐type combustion C–N elemental analyzer (Vario MAX CN; Elementar Analysensysteme GmbH, Hanau, Germany). Leaf P (mg/g) and K (mg/g) concentrations were analyzed using an inductively coupled plasma atomic emission spectrometer (iCAP 7400; Thermo Fisher Scientific, Bremen, Germany). C:N, C:P, and N:P ratios were calculated as the indicators of plant nutrient use efficiencies and limitations (Elser, Sterner, et al. 2000).

### 2.4 Data Analyses

Leaf trait data were averaged across five individual trees per rocky desertification category and log_10_‐transformed to enhance distribution normality. Differences in leaf traits between forests with varying degrees of rocky desertification were assessed using a one‐way analysis of variance. Pearson's correlation analysis was used to evaluate relationships between leaf traits. Principal component analysis (PCA) was performed to explore trait associations. All statistical analyses were performed using R version 4.4.0 (R Core Team 2024).

## 3 Results

### 3.1 Differences in Leaf Morphological and Anatomical Traits

**FIGURE 2:**
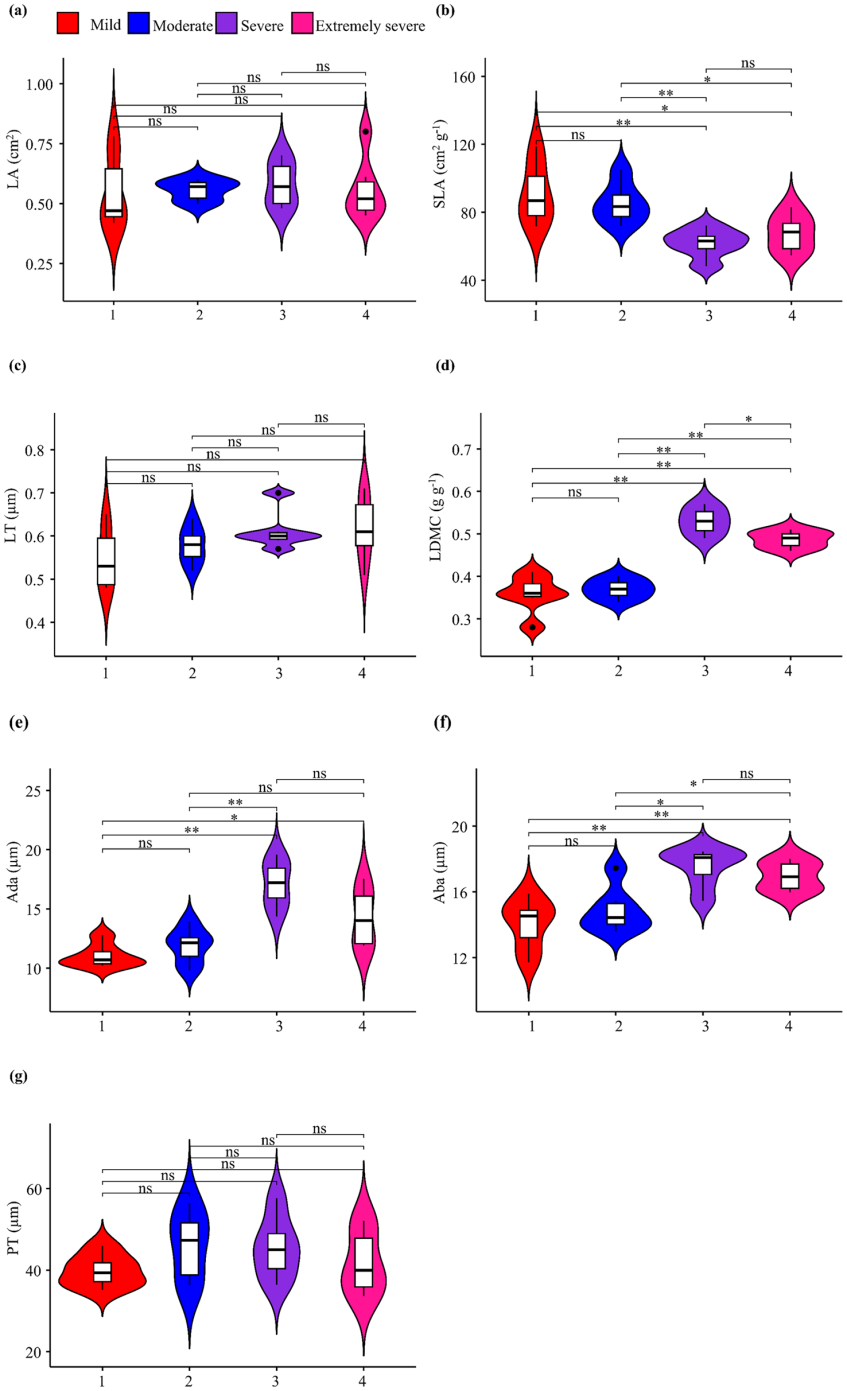
Variation in leaf morphological and anatomical traits of *Pseudotsuga sinensis* across forests with different degrees of rocky desertification. Aba, abaxial epidermis thickness; Ada, adaxial epidermis thickness; LA, leaf area; LDMC, leaf dry matter content; LT, leaf thickness; PT, palisade mesophyll thickness; SLA, specific LA. *, *p* < 0.05; **, *p* < 0.01; ns, *p* > 0.05.

The SLA of *P. sinensis* leaves was significantly greater in forests with mild or moderate rocky desertification than in those with severe (vs. mild, *p* < 0.01; vs. moderate, *p* < 0.01) or extremely severe (vs. mild, *p* < 0.05; vs. moderate, *p* < 0.05) rocky desertification (Figure 2b). However, there were no significant differences in SLA between forests with mild or moderate desertification or between forests with severe or extremely severe desertification (Figure 2b; Table 2). LDMC was significantly higher in forests with severe (vs. mild, *p* < 0.01; vs. moderate, *p* < 0.01) or extremely severe (vs. mild, *p* < 0.01; vs. moderate, *p* < 0.01) rocky desertification than in those with mild or moderate desertification (Figure 2d). Moreover, LDMC was also significantly higher in forests with extremely severe desertification than in those with severe desertification. No significant differences in LDMC were observed between forests with mild or moderate desertification (Figure 2d). Ada was significantly greater in forests with severe (vs. mild, *p* < 0.01) or extremely severe (vs. mild, *p* < 0.05) rocky desertification than in those with mild desertification (Figure 2e). Moreover, Ada was also significantly higher in forests with severe desertification than in those with moderate desertification. However, no significant differences in Ada were found between forests with mild or moderate desertification, between those with severe or extremely severe desertification, or between those with severe or moderate desertification (Figure 2e). Aba was significantly higher in forests with severe (vs. mild, *p* < 0.01; vs. moderate, *p* < 0.05) or extremely severe (vs. mild, *p* < 0.01; vs. moderate, *p* < 0.05) rocky desertification than in those with mild or moderate desertification, with no significant differences between forests with mild or moderate or between those with severe or extremely severe desertification (Figure 2f). No significant differences were observed in LA, LT, or PT between these forests with four varying degrees of rocky desertification (Figure 2a,c,g).

**TABLE 2:** Differences in the leaf traits of *Pseudotsuga sinensis* in forests with different degrees of rocky desertification (mean ± standard error).

Traits	Mild rocky desertification	CV (%)	Moderate rocky desertification	CV (%)	Severe rocky desertification	CV (%)	Extremely severe rocky desertification	CV (%)
LA (cm^2^)	0.55 ± 0.06	25.58	0.56 ± 0.02	7.59	0.58 ± 0.04	16.17	0.56 ± 0.48	23.36
SLA (cm^2^ g^−1^)	90.85 ± 6.69	18.04	85.26 ± 4.43	13.96	61.71 ± 3.08	13.41	67.44 ± 4.07	16.19
LT (μm)	0.55 ± 0.03	11.79	0.58 ± 0.02	7.45	0.61 ± 0.02	7.41	0.62 ± 0.03	12.12
LDMC (g g^−1^)	0.36 ± 0.02	11.33	0.37 ± 0.01	6.16	0.53 ± 0.74	5.97	0.49 ± 0.01	4.04
Ada (μm)	11.05 ± 0.37	8.14	11.89 ± 0.55	12.47	17.11 ± 0.44	11.64	14.27 ± 0.92	17.28
Aba (μm)	14.07 ± 0.57	9.84	14.90 ± 0.52	9.45	17.52 ± 2.87	6.76	16.96 ± 0.33	5.23
PT (μm)	39.80 ± 1.47	9.04	46.06 ± 3.08	17.95	45.52 ± 0.91	16.93	41.78 ± 2.90	18.64
C (mg g^−1^)	486.29 ± 1.94	0.98	485.47 ± 3.19	1.76	490.51 ± 2.87	0.50	492.64 ± 1.84	0.99
N (mg g^−1^)	14.07 ± 0.29	5.09	13.66 ± 0.64	12.46	13.27 ± 0.31	6.30	13.78 ± 0.49	9.57
P (mg g^−1^)	1.42 ± 0.15	25.54	1.66 ± 0.12	19.90	1.85 ± 0.11	16.47	1.45 ± 0.21	39.01
K (mg g^−1^)	5.88 ± 0.39	16.02	4.88 ± 0.55	30.41	5.22 ± 0.26	13.08	4.01 ± 0.40	26.70
Ca (mg g^−1^)	6.70 ± 0.40	14.64	6.47 ± 0.60	24.92	10.28 ± 1.01	26.46	8.26 ± 1.04	33.88
Mg (mg g^−1^)	1.76 ± 0.10	14.53	2.13 ± 0.18	22.49	0.98 ± 0.04	11.85	1.11 ± 0.06	15.47
C:N	34.65 ± 0.65	4.60	35.93 ± 1.43	10.65	37.10 ± 0.89	6.43	35.99 ± 1.16	8.67
C:P	366.81 ± 39.25	26.21	322.70 ± 33.35	21.66	298.93 ± 34.48	15.67	265.89 ± 13.00	34.82
N:P	10.55 ± 1.04	24.02	8.44 ± 0.64	20.31	7.31 ± 0.38	14.00	10.53 ± 1.23	31.23

Abbreviations: Aba, abaxial epidermis thickness; Ada, adaxial epidermis thickness; CV, coefficient of variation; LA, leaf area; LDMC, leaf dry matter content; LT, leaf thickness; PT, palisade mesophyll thickness; SLA, specific LA.

### 3.2 Differences in Leaf Nutrient Contents

**FIGURE 3:**
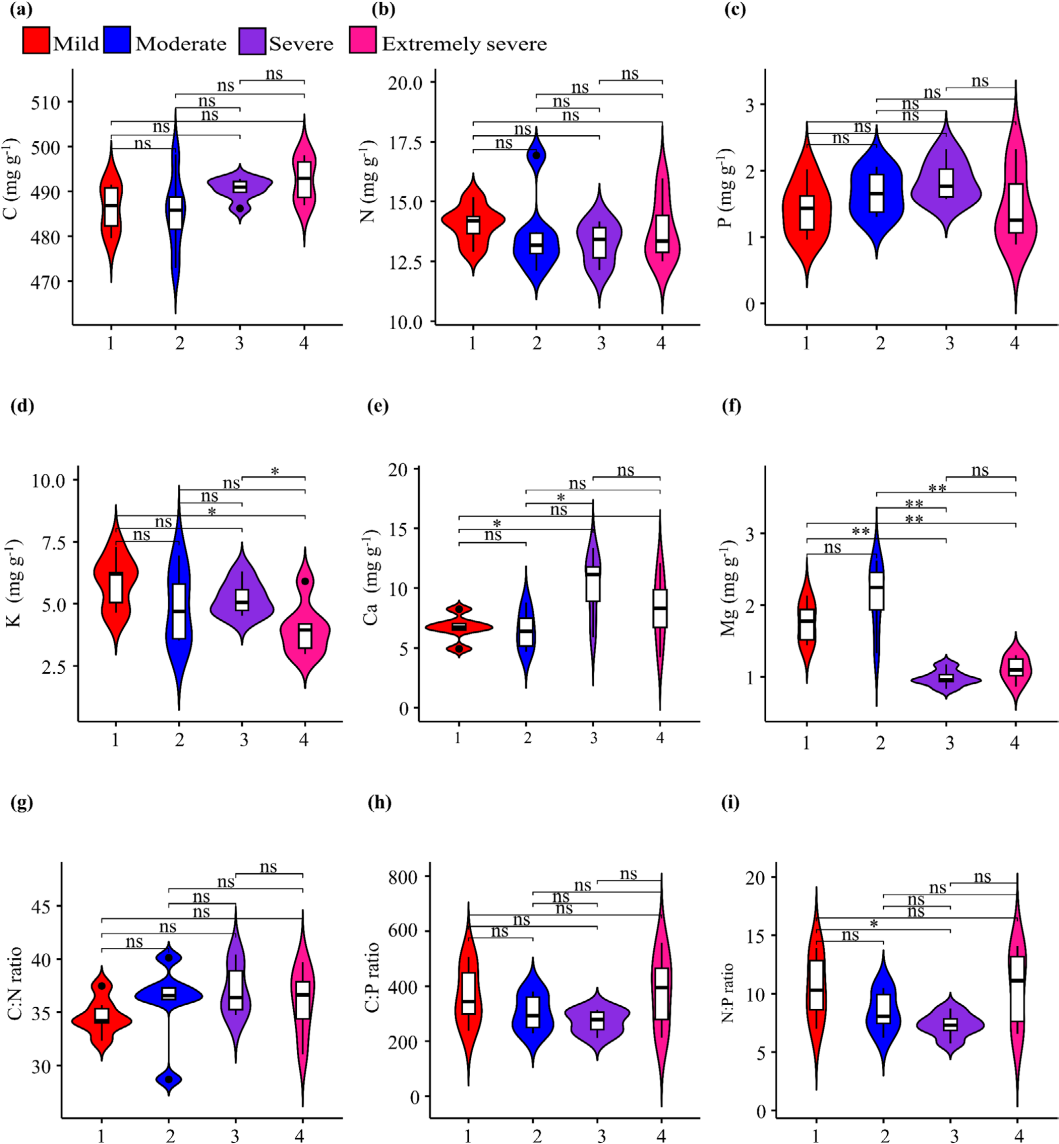
Variation in the leaf nutrient concentrations and stoichiometric characteristics of *Pseudotsuga sinensis* across forests with varying levels of rocky desertification. *, *p* < 0.05; **, *p* < 0.01; ns, *p* > 0.05.

Regarding leaf nutrient contents and their stoichiometric characteristics, K concentrations in *P. sinensis* leaves were significantly higher in forests with mild or severe rocky desertification than in those with extremely severe rocky desertification. However, K concentrations did not differ significantly between forests with mild, moderate, or severe rocky desertification or between forests with severe or extremely severe desertification (Figure 3d; Table 2). Leaf Ca concentrations were significantly higher in forests with severe rocky desertification than in those with mild or moderate desertification but did not differ significantly between forests with moderate, severe, or extremely severe or between forests with mild or moderate desertification (Figure 3e). Leaf Mg concentrations were significantly higher in forests with mild or moderate rocky desertification than in those with severe or extremely severe desertification but did not differ significantly between forests with mild or moderate or between forests with severe or extremely severe desertification (Figure 3f). The N:P ratio was significantly higher in forests with mild rocky desertification than in those with severe desertification but did not differ significantly between forests with mild, moderate, or extremely severe rocky desertification (Figure 3i). There were no significant differences in leaf C, N, and P contents and C:N and C:P ratios between these forests with four different degrees of rocky desertification (Figure 3a–c,g,h).

### 3.3 Pearson's Correlations Between Leaf Traits

**FIGURE 4:**
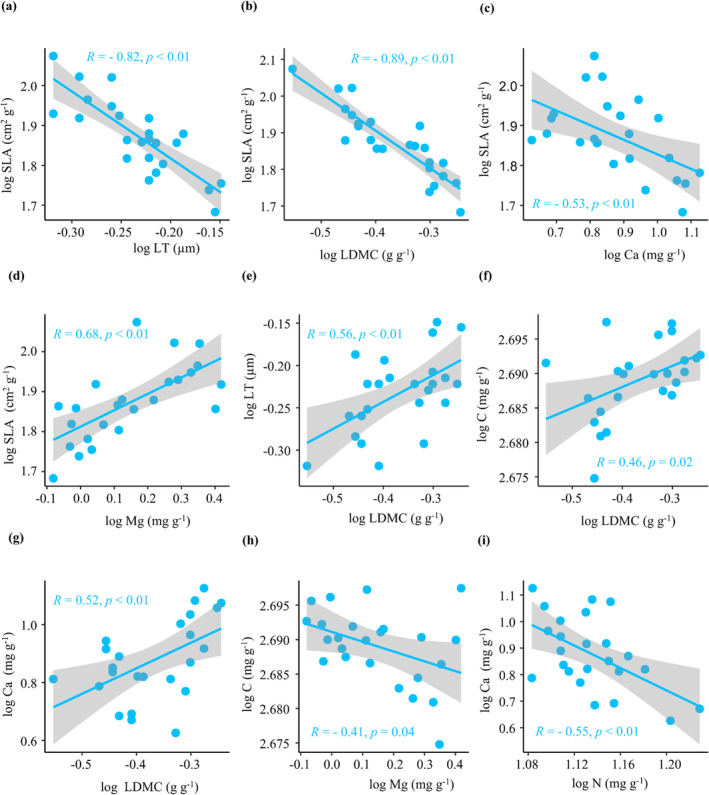
Pearson's correlations between the leaf functional traits of *Pseudotsuga sinensis* in forests with different degrees of rocky desertification. LDMC, leaf dry matter content; LT, leaf thickness; SLA, specific leaf area.

Among all leaf traits, SLA correlated negatively with LT, LDMC, and leaf Ca concentrations (Figure 4a–c) and positively with leaf Mg concentrations (Figure 4d). LDMC correlated positively with LT and leaf C and Ca concentrations (Figure 4e–g), whereas leaf Mg concentrations correlated negatively with leaf C concentrations (Figure 4h). Leaf N concentrations correlated negatively with leaf Ca concentrations (Figure 4i).

### 3.4 Leaf Trait Associations

**FIGURE 5:**
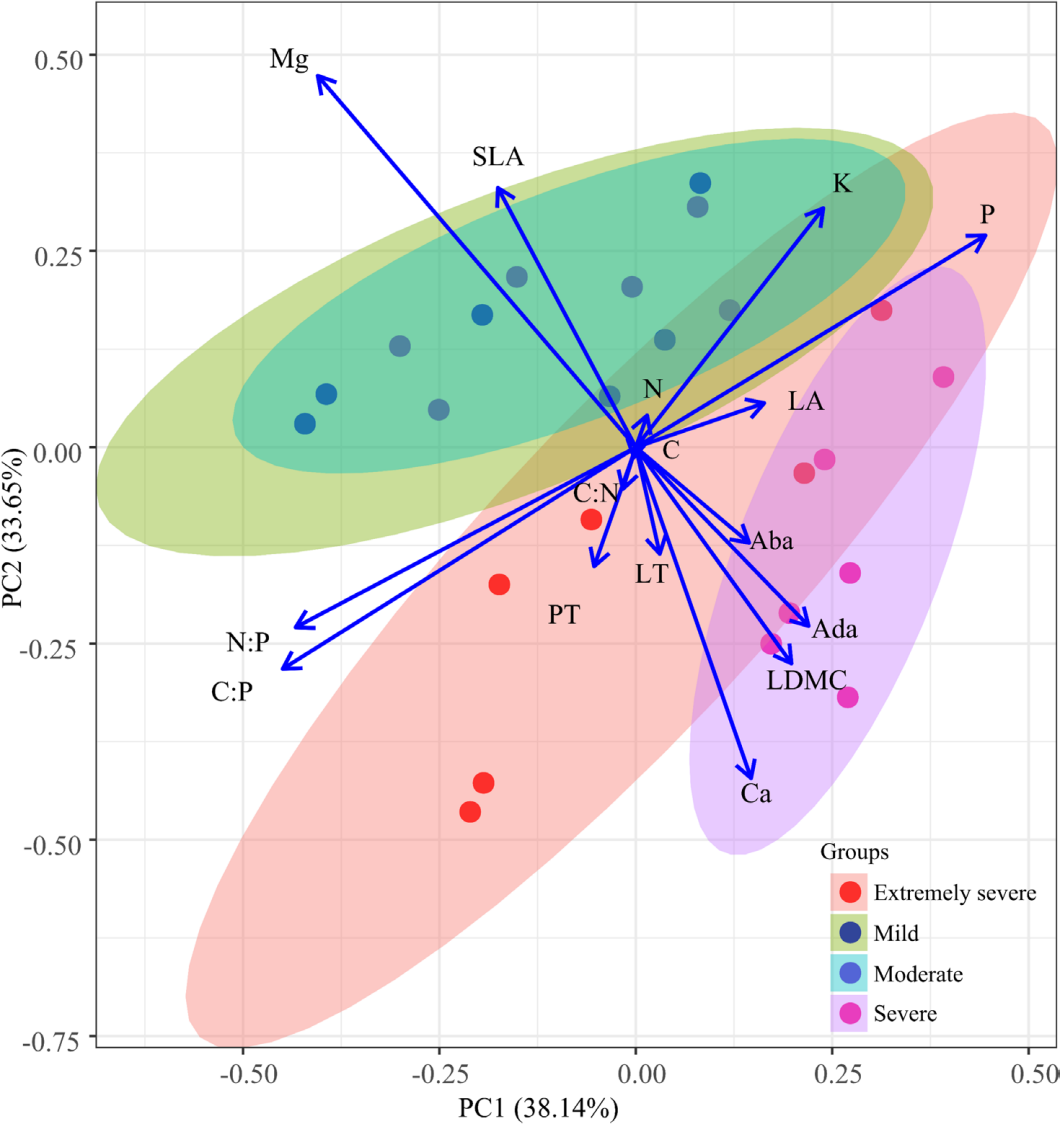
Biplot of the first two principal components (PC1 and PC2) illustrating the relationships between different leaf functional traits and their loadings across forests with varying degrees of rocky desertification. All leaf traits were log_10_‐transformed prior to analysis. Aba, abaxial epidermis thickness; Ada, adaxial epidermis thickness; LA, leaf area; LDMC, leaf dry matter content; LT, leaf thickness; PT, palisade mesophyll thickness; SLA, specific LA.

The results of the PCA of the 16 leaf traits of leaves from 20 *P. sinensis* individual trees revealed that the first and second components explained 38.14% and 33.65% of the total variance, respectively (Figure 5). The first principal component correlated positively with LA and leaf P and K concentrations and negatively with C:N, C:P, and N:P ratios. The second principal component correlated positively with SLA and leaf Mg concentrations and negatively with LDMC, LT, PT, Ada, Aba, and leaf Ca concentrations. Forests with mild or moderate rocky desertification correlated positively with SLA and leaf Mg concentrations, indicating a resource acquisition strategy. In contrast, forests with severe rocky desertification correlated positively with leaf P and K concentrations but negatively with C:N, C:P, and N:P ratios, suggesting a resource conservation strategy. Forests with extremely severe rocky desertification showed a positive correlation with Aba, Ada, LDMC, LT, and PT but a negative correlation with SLA and leaf Mg concentrations, indicating a resource conservation strategy (Figure 5).

## 4 Discussion

### 4.1 Differences in Leaf Morphological and Anatomical Traits

Functional traits often reflect the adaptation of plants to their environments (Knight and Ackerly 2003; Burns 2004; Zhang et al. 2020; Islam et al. 2024). The results obtained in our study revealed that SLA decreased as the degree of rocky desertification increased, whereas LDMC, Ada, and Aba increased with increasing degrees of rocky desertification. Previous research has shown that SLA is a key indicator of plant responses to environmental stress, typically decreasing in resource‐poor environments (Reich et al. 1998; Xiao et al. 2022). A lower‐than‐normal SLA can help minimize water loss during metabolic activities (Wright et al. 2005). Conversely, LDMC, which indicates leaf nutrient retention capacity, is generally higher in resource‐limited habitats than in resource‐rich habitats (Cornelissen et al. 2003; Yang et al. 2014). Plants in harsh environments often develop thicker leaves and tissues to enhance water storage and utilization efficiency than those in favorable environments (Liu, Liu, and Guo 2011; Zhang et al. 2019; Zhang et al. 2020). Our findings suggest that *P. sinensis* adapts to increasing levels of rocky desertification by conserving resources through reduced SLA, increased LDMC, and increased leaf tissue thickness, thereby improving its resilience to arid conditions.

### 4.2 Differences in Leaf Nutrient Contents

Leaf C concentrations in *P. sinensis* across all levels of rocky desertification, that is, mild (486.29 mg g^−1^), moderate (485.47 mg g^−1^), severe (490.51 mg g^−1^), and extremely severe (492.64 mg g^−1^), in our study exceeded the reported global average for terrestrial plant species (464.0 mg g^−1^; Elser, Fagan, et al. 2000). Additionally, in our study, *P. sinensis* showed higher leaf C concentrations than those reported for *Pinus dabeshanensis* (434.54 mg g^−1^; Zhou 2023) and *Pinus yunnanensis* (441.42 mg g^−1^; Liu and Dong 2020), indicating a superior C storage capacity in *P. sinensis* across the varying degrees of rocky desertification. Leaf N concentrations in *P. sinensis* in our study were lower than the reported average for Chinese terrestrial plants (18.6 mg g^−1^; Han et al. 2005) but were higher than those in other species in the same family, such as *Pinus yunnanensis* (4.63 mg g^−1^; Liu and Dong 2020) and *Pinus armandii* (10.84 mg g^−1^; Dong et al. 2017). This finding suggests that despite the relatively high leaf N concentrations in *P. sinensis*, their growth in subtropical high‐altitude forests, which is typically N‐limited (Yin et al. 2022), may remain constrained by N availability. Leaf P concentrations in *P. sinensis* from forests with varying degrees of rocky desertification, that is, mild (1.42 mg g^−1^), moderate (1.66 mg g^−1^), severe (1.85 mg g^−1^), and extremely severe (1.45 mg g^−1^), in our study, were lower than the reported global average for terrestrial plants (1.99 mg g^−1^; Elser, Fagan, et al. 2000) but higher than those in several other species in the same family, including *Pinus dabeshanensis* (1.23 mg g^−1^; Zhou 2023), *Pinus yunnanensis* (0.80 mg g^−1^; Liu and Dong 2020), and *Pinus armandii* (1.42 mg g^−1^; Dong et al. 2017). This result aligns with previous findings that P concentrations in Chinese terrestrial plants are generally lower than the reported global average (Han et al. 2005), possibly because of P deficiency in areas with karst rocky desertification (Wu 2017; Wen, Wang, and Sheng 2018). Leaf K and Mg concentrations in *P. sinensis* decreased with increasing degrees of rocky desertification. This trend may be attributed to increased soil erosion and reduced soil nutrient content available for plant uptake (Xiong, Li, and Long 2012). Conversely, Ca concentrations in leaves increased with increasing degrees of rocky desertification. This increase may have resulted from the variable chemical dissolution rates of soluble carbonate rocks in areas with different degrees of desertification, affecting both soil and plant Ca concentrations (Ji, Li, and Deng 2009; Xiong, Li, and Long 2012). The N:P ratio, an indicator of nutrient limitation, was lower than 14 across all levels of rocky desertification (mild: 10.55; moderate: 8.44; severe: 7.31; and extremely severe: 10.53), suggesting that the growth of *P. sinensis* is predominantly limited by N in these environments, which is consistent with the findings of previous studies (Koerselman and Meuleman 1996; Elser, Sterner, et al. 2000; Güsewell 2004).

### 4.3 Leaf Trait Associations

During their growth and development, plants develop a series of optimal combinations of functional traits to adapt to environmental changes (Westoby et al. 2002; Wright et al. 2007; Ahrens et al. 2020; Maynard et al. 2022). In the present study, we found that resource acquisition traits (SLA and leaf Mg and N concentrations) correlated significantly negatively with resource conservation traits (LDMC, LT, and leaf C and Ca concentrations). In arid and poor soil environments, increased levels of leaf photosynthates are used to build protective tissues or increase mesophyll density to prevent leaf damage or water loss from high temperatures, thus improving water‐use efficiency (Cornelissen et al. 2003; Yang et al. 2014; Islam et al. 2024). Previous studies have reported a negative correlation between the SLA and LDMC of the leaves of *Pinus massoniana* . Similar trade‐offs have been reported in other studies on forests with karst rocky desertification. For example, studies on forests with karst rocky desertification reported that SLA correlates negatively with LT and LDMC in such forests (Zhong et al. 2018; Xiong et al. 2022). Our results indicate that *P. sinensis* has evolved a set of trait combinations suitable for arid habitats after long‐term environmental screening in areas with rocky desertification.

Collectively, our results revealed significant differences in the leaf traits of *P. sinensis* across forests with varying degrees of rocky desertification. Specifically, traits such as SLA, LDMC, Ada, Aba, leaf K, Ca, and Mg concentrations, and the N:P ratio varied significantly with variations in the degree of rocky desertification. These variations suggest that different adaptive strategies are employed by *P. sinensis* depending on the severity of desertification. Thus, it can be inferred that *P. sinensis* exhibits a resource acquisition strategy in forests with mild or moderate rocky desertification, as evidenced by higher SLA and leaf K concentrations than those in forests with severe or extremely severe rocky desertification, reflecting a strategy that favors resource acquisition in relatively less stressed environments. Conversely, in forests undergoing severe and extremely severe rocky desertification, this species adopts a resource conservation strategy, as indicated by increased LDMC, Ada, Aba, and leaf Ca and Mg concentrations, highlighting adaptations to relatively more stressful conditions. The different adaptive strategies of *P. sinensis* under different degrees of rocky desertification provide new insights for developing measures for its protection and restoration. For mild or moderate rocky desertification areas, our efforts must be focused on ensuring essential nutrient and water conditions for *P. sinensis* to maintain its dominant position within this ecosystem and the stability of the entire ecosystem. For severe or extremely severe rocky desertification areas, relatively more intensive interventions need to be carried out, such as artificially introducing species with similar adaptation strategies (resource‐conservation strategies) to improve species diversity and reform the conditions of such degraded habitats.

## 5 Conclusions

In this study, we evaluated 16 leaf traits of *P. sinensis* across forests with varying degrees of rocky desertification. Our findings revealed that SLA and leaf Mg and K concentrations in *P. sinensis* decreased with increasing severity of rocky desertification. Conversely, LDMC, Ada, Aba, and leaf Ca concentrations increased with increasing degrees of desertification, indicating a shift in the adaptive strategy of *P. sinensis* from a resource acquisition strategy to a resource conservation strategy as rocky desertification intensifies. Additionally, N appeared to be a limiting factor for the growth of *P. sinensis* in regions with rocky desertification. These results will provide valuable reference for guiding vegetation restoration under degraded habitats.

In future studies, we will comprehensively analyze and explore the adaptation mechanisms and limiting factors of *P. sinensis* distribution with respect to soil nutrient contents, stem and root morphological traits and nutrient contents, litter nutrient contents, soil microorganisms, and leaf photosynthesis rates.

## Author Contributions

**Wangjun Li:** investigation (equal), methodology (equal), writing – original draft (lead), writing – review and editing (equal). **Wanchang Zhang:** writing – review and editing (lead). **Tu Feng:** investigation (equal), methodology (equal). **Dongpeng Lv:** methodology (equal). **Shun Zou:** investigation (equal). **Bin He:** investigation (equal). **Xiaolong Bai:** conceptualization (equal), methodology (equal), writing – review and editing (equal).

## Conflicts of Interest

The authors declare no conflicts of interest.

## Data Availability

The data that support the findings of this study are openly available in the Dryad data repository at https://doi.org/10.5061/dryad.zcrjdfnnw, with a reviewer URL of https://datadryad.org/stash/share/t9BS9ey0zSYnFiS3mfBLfOfS4APYw_5zBdZLnLJaaM4.
